# Towards a needle-free diagnosis of malaria: in vivo identification and classification of red and white blood cells containing haemozoin

**DOI:** 10.1186/s12936-017-2096-1

**Published:** 2017-11-07

**Authors:** Jennifer L. Burnett, Jennifer L. Carns, Rebecca Richards-Kortum

**Affiliations:** Department of Bioengineering, Rice University, 6100 Main Street, Houston, TX 77005 USA

**Keywords:** Malaria diagnostics, In vivo, Microscopy, Haemozoin, Pigment-containing white blood cells

## Abstract

**Background:**

Optical detection of circulating haemozoin has been suggested as a needle free method to diagnose malaria using in vivo microscopy. Haemozoin is generated within infected red blood cells by the malaria parasite, serving as a highly specific, endogenous biomarker of malaria. However, phagocytosis of haemozoin by white blood cells which persist after the infection is resolved presents the potential for false positive diagnosis; therefore, the focus of this work is to identify a feature of the haemozoin signal to discriminate between infected red blood cells and haemozoin-containing white blood cells.

**Methods:**

Conventional brightfield microscopy of thin film blood smears was used to analyse haemozoin absorbance signal in vitro. Cell type and parasite maturity were morphologically determined using colocalized DAPI staining. The ability of features to discriminate between infected red blood cells and haemozoin-containing white blood cells was evaluated using images of smears from subjects infected with two species of *Plasmodium*, *Plasmodium yoelii* and *Plasmodium falciparum*. Discriminating features identified by blood smear microscopy were characterized in vivo in *P. yoelii*-infected mice.

**Results:**

Two features of the haemozoin signal, haemozoin diameter and normalized intensity difference, were identified as potential parameters to differentiate infected red blood cells and haemozoin-containing white blood cells. Classification performance was evaluated using the area under the receiver operating characteristic curve, with area under the curve values of 0.89 for the diameter parameter and 0.85 for the intensity parameter when assessed in *P. yoelii* samples. Similar results were obtained from *P. falciparum* blood smears, showing an AUC of 0.93 or greater for both classification features. For in vivo investigations, the intensity-based metric was the best classifier, with an AUC of 0.91.

**Conclusions:**

This work demonstrates that size and intensity features of haemozoin absorbance signal collected by in vivo microscopy are effective classification metrics to discriminate infected red blood cells from haemozoin-containing white blood cells. This reduces the potential for false positive results associated with optical imaging strategies for in vivo diagnosis of malaria based on the endogenous biomarker haemozoin.

**Electronic supplementary material:**

The online version of this article (10.1186/s12936-017-2096-1) contains supplementary material, which is available to authorized users.

## Background

Haemozoin (Hz), also referred to as the malaria pigment, is generated within infected red blood cells (iRBCs) by the malaria parasite. Due to its unique optical properties, Hz serves as an endogenous biomarker that has been explored in several in vivo malaria diagnostic approaches [1–3]; however, Hz is also found in white blood cells that have phagocytized either iRBCs or free Hz following iRBC rupture. The concurrent presence of pigment-containing white blood cells (pWBCs) may inflate the quantification of iRBCs leading to over estimation of parasitaemia with optical imaging methods. Concentrations of pWBCs measured in peripheral blood smears have been reported in several studies [4–8] and are summarized in Additional file 1: Table S1. Average concentrations of pigment-containing neutrophils and pigment-containing monocytes are 267/µl (range 0–3721) and 95/µl (range 0–3420) respectively. The large range of these values may be attributed to factors such as age of the study cohort, disease severity, and acquired immunity. In all cases, parasitaemia ranged from 1 to 3 orders of magnitude greater than the concentration of pWBCs per microlitre. Also, pWBCs may persist days after iRBC clearance and resolution of the infection [6], thereby presenting the potential for false positive diagnosis. To avoid misinterpreting Hz detected in pWBCs as iRBCs, it is critical that diagnostic methods relying on Hz detection differentiate these two cell types.

Detecting and quantifying pWBCs has clinical relevance beyond avoiding false positive diagnoses. Several studies have correlated the concentration of pWBCs in blood smears with disease severity in both adults and children [8–10], and in some cases have determined pWBC concentration to be a better indicator of disease severity than parasite density [5, 11]. The presence of pWBCs has also been identified as a marker of sequestered infection and a better measure of total parasite biomass than peripheral blood parasite density [7, 8, 12]. Pregnancy-associated malaria, currently diagnosed by examination of the placenta after birth, is one highly studied form of sequestration and is associated with poor health outcomes for both mother and baby [13]. A study following gestational malaria cases found the presence of pWBCs, specifically neutrophils, in peripheral blood smears to be a positive prognostic marker for decreased birth weight [14]. These findings suggest that the identification of pWBCs could further the clinical utility of a Hz-based diagnostic method.

In this paper, an analytical method using Hz absorbance to discriminate between two sources of Hz signal, iRBC and pWBC, is introduced. A method to detect malaria in vivo by targeting the Hz absorbance peak centered at 655 nm was recently developed [1]. This approach uses a microscope to optically detect Hz circulating in the superficial vasculature. One of the advantages of using Hz as a biomarker is that it does not require the application of an exogenous contrast agent for visualization. Here, features of the Hz absorbance signal are assessed using blood smear microscopy analysis of *Plasmodium yoelii*-infected murine blood samples. Good discrimination between iRBCs and pWBCs was observed using a size-based feature, Hz effective diameter, and an intensity-based feature, relative intensity difference. This approach was further evaluated in vivo using mice with either active or recent *P. yoelii* infections. Images of circulating Hz were collected by in vivo microscopy and analysed using the same two discriminating features assessed by blood smear microscopy. Both features accurately classified cells as iRBCs or pWBCs, with the intensity-based discriminator having the greatest accuracy. These two discriminating features were then evaluated by blood smear microscopy using patient-derived and cultured *Plasmodium falciparum* blood samples. Discrimination between the two cell types was more accurate for *P. falciparum*, likely due to the absence of mature iRBCs which sequester in *P. falciparum* infections. These results suggest that this approach may be used to identify the presence of both iRBCs and pWBCs and to discriminate between active infections and recent infections, furthering the clinical utility of needle-free Hz-based malaria diagnosis.

## Methods

### Blood smear sample preparation

Smears were prepared from blood infected with two different species of *Plasmodium*, the rodent-infecting species *P. yoelii* and the human-infecting species *P. falciparum. Plasmodium yoelii* preferentially infects reticulocytes, similar to *Plasmodium vivax*, the predominant human-infecting species causing recurring malaria [15]. Blood from *P. yoelii*-infected mice was collected by cardiac puncture into heparinized tubes and used to prepare thin film smears. Unstained and unfixed thin film blood smears infected with *P. falciparum* at 0.5% parasitaemia were acquired from McGill University J.D. MacLean Centre for Tropical Diseases. Negative control smears were prepared from blood acquired from healthy normal volunteers. All smears were fixed in methanol and allowed to dry. A drop of liquid mounting medium containing DAPI fluorescent nuclear stain (Invitrogen P-36931) was applied to each smear prior to sealing with a coverslip. DAPI has been previously demonstrated as an effective contrast agent for parasite nuclei within infected red blood cells and for white blood cell differentiation [16]. Additionally, the DAPI absorbance peak, centered at 358 nm, does not interfere with Hz absorbance measurements.

### In vitro phagocytosis of cultured *Plasmodium falciparum* lysate

To simulate WBC phagocytosis of Hz, isolated Hz was added to whole blood from a healthy volunteer using methods adapted from Frita et al. [17]. Hz was isolated from *P. falciparum* culture (Strain 3D7, acquired from PATH, Seattle, Washington). The cultured sample was washed with 1× phosphate buffered saline to remove glycerin and resuspended at 5% haematocrit, 5% parasitaemia. To induce RBC lysis, the sample was centrifuged for 10 min at 1000 *g*, resuspended in 1 ml of 0.15% saponin (Sigma), and incubated for 10 min on ice with intermittent vortexing. Following incubation, the sample was washed twice with phosphate buffered saline at 10,000 g for 15 min. The final isolated Hz pellet was resuspended in 1 ml of phosphate buffered saline.

Whole blood from a healthy volunteer was collected into heparinized tubes and diluted 1:1 in cell culture media (Sigma, RPMI 1640) at 10% FBS. Isolated Hz was added to the diluted blood 1:1 and the mixture was plated into a 48 well plate and incubated at 37 °C at 5% CO_2_. After 4 h of incubation, samples were used to prepare thin film blood smears for analysis. A DAPI-containing mounting medium was applied to the smears before sealing with a coverslip.

### Blood smear image acquisition and analysis

Images of blood smears were acquired with an upright conventional microscope (Zeiss, Z1) using a 63× oil immersion objective. Two images were collected from each field of view, the first under brightfield transmission illumination and the second using a DAPI fluorescence filter set (Zeiss, Filter Set 49). For brightfield imaging, light from a broadband halogen lamp was passed through a narrow bandpass filter (Chroma, HQ 655/40) to isolate Hz absorbance and minimize haemoglobin Q-band absorbance.

**Fig. 1:**
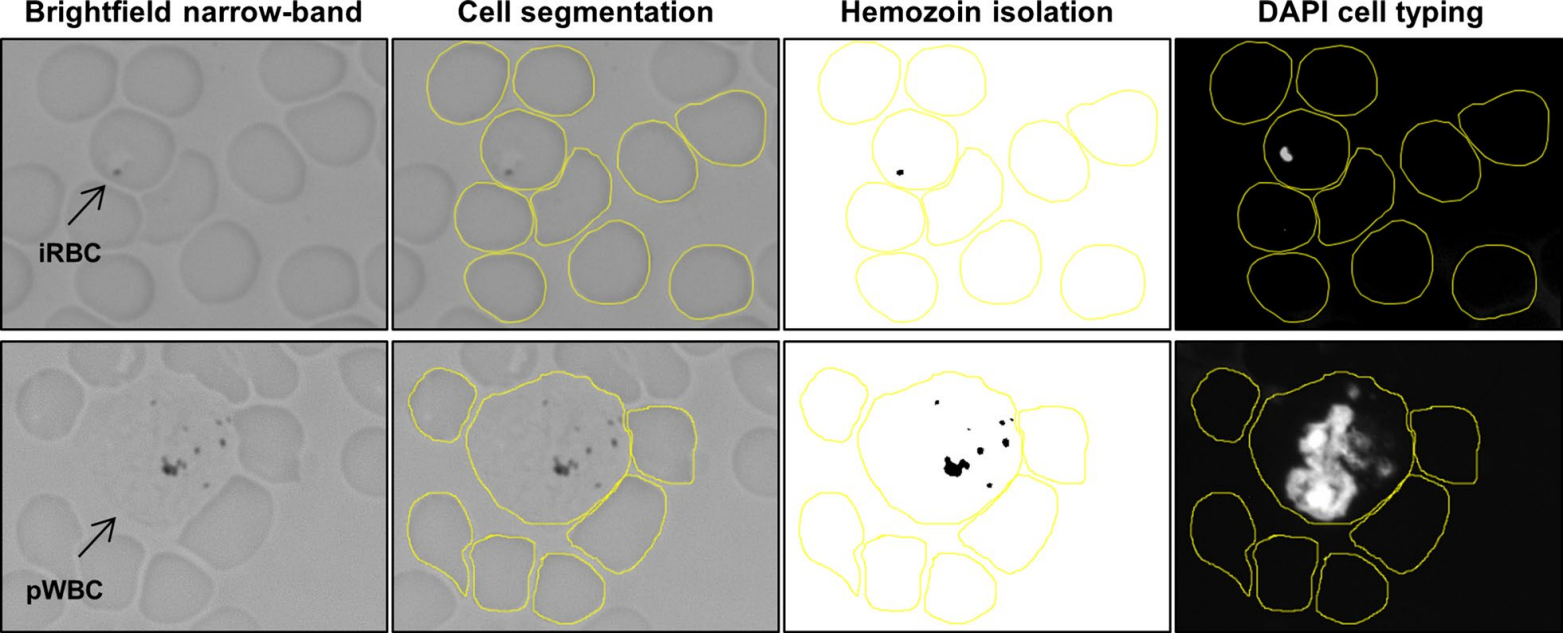
Blood smear image analysis. Images of thin-film blood smears containing a *Plasmodium falciparum* iRBC (top row) and a pWBC (bottom row), both surrounded by uninfected RBCs. Brightfield images were collected under narrowband illumination (λ = 655 nm ± 20 nm). Cells were segmented using the brightfield image. An intensity threshold was applied to isolate the haemozoin absorbance signal. DAPI staining was used to identify cell type

Images were analysed using the open source platform ImageJ (National Institutes of Health, v1.47t), following the process illustrated in Fig. 1. Individual red and white blood cells were manually segmented using the brightfield images. A background intensity threshold (I_T_), set as the average minimum brightfield pixel intensity of uninfected red blood cells, was applied to brightfield images to isolate Hz signal. Cell type was determined by visual examination of morphology in the corresponding DAPI image. WBCs were identified on the basis of their large nucleus observed under DAPI staining. RBCs have no DAPI signal, whereas parasite nuclei can be clearly distinguished in iRBCs. Parasite maturity was also determined using the DAPI signal, where iRBCs containing only a single parasite nucleus were identified as young trophozoites. Multinucleated iRBCs were labelled as late trophozoites. As a negative control, the same process was repeated for RBCs and WBCs in blood smears from non-infected mice and uninfected healthy volunteer human subjects.

Hz area and mean pixel intensity were recorded for each segmented cell. These data were used to calculate the Hz effective diameter (d_eff_) and relative intensity difference (RID), two parameters assessed for their ability to discriminate between iRBCs and pWBCs. The Hz d_eff_ was estimated from the measured Hz area (A), where d_eff_ = 2 (A/π)^1/2^. RID was measured as the percent difference between the background threshold intensity and the mean Hz pixel intensity, expressed as RID = (I_T_ − <I_HZ_>)/I_T_. Hz d_eff_ and RID were plotted for all cells versus cell type using the DAPI-determined cell type. Box plots characterizing the first quartile, median, and third quartile were generated for each cell type. Whiskers of these plots extend to ± 1.58 (IQR/(n^1/2^)), where IQR is the interquartile range and n is the number of observations. Mann–Whitney rank sum tests were performed to measure statistical differences between median values of the cell types. Linear discriminant analysis was used to develop an algorithm to classify iRBCs and pWBCs based on the Hz d_eff_ and the RID; results were compared to the DAPI-determined cell type and the resulting area under the ROC curve was plotted.

To address the effect of the relative ratio of iRBC to pWBC on the AUC, the AUC was measured for datasets with an iRBC:pWBC relative ratio of 1:10. To achieve this, the iRBC dataset was randomly subdivided into ten equal-sized subsets. The AUC was measured for each iRBC subset compared to the full pWBC data set, giving a range of AUC depending on the data subset measured. This same process was repeated for ten datasets with an iRBC:pWBC relative ratio of 10:1.

### In vivo assessment of haemozoin classification algorithm

**Fig. 2:**
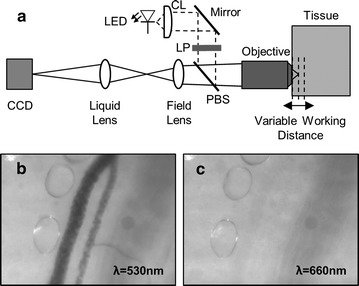
In vivo imaging using the microvascular microscope. **a** The microvascular microscope (MvM) was used for in vivo investigations to detect haemozoin circulating in the superficial microvasculature. The MvM employs cross polarized epi-illumination to optically access vessels below the tissue surface. LED illumination is collimated by an achromatic collimating lens (CL), polarized using a linear polarizer (LP) followed by a collinear polarizing beam splitter (PBS), and finally delivered to the tissue through a microscope objective. Diffusely scattered depolarized light is collected by the microscope objective and imaged onto a CCD detector. An example field of view from an uninfected subject is shown in the bottom row. Vessels were located under green light illumination (**b**) and red light illumination was used to detect haemozoin circulating through the vessel (**c**)

The ability to discriminate between iRBCs and pWBCs in vivo was evaluated in a mouse model of malaria. Three cohorts of mice—negative controls (n = 3), acute infection (n = 4), and post infection (n = 3)—were included in the in vivo assessment. Female albino B6 mice were acquired from Jackson Laboratories at 7 weeks of age. Malaria infection was generated by i.p. inoculation of *P. yoelii* XNL (MRA-593, MR4, ATCC Manassas Virginia). Mice with acute infections, i.e. without pWBC present in the blood, were used to assess Hz signal originating from iRBCs. Mice were imaged post infection to measure Hz signal originating from pWBCs only. The surgically accessed reflected dorsal skin flap was used to mimic the tissue environment of a human mucous membrane. Hz signal was detected using a microvascular microscope (MvM), a brightfield microscope that was previously demonstrated to detect Hz in vivo and is described elsewhere [1]. For these investigations, cross polarized epi-illumination was used to optically access vessels below the tissue surface. A block diagram of the MvM and representative images collected using a non-infected mouse are shown in Fig. 2. The MvM was brought into gentle contact with the exposed vasculature of the skin flap for image collection. Linearly polarized light was delivered to the tissue by epi-illumination. Specular reflection was rejected by a polarizing beam splitter (Thorlabs, PBS201). Diffusely scattered depolarized light was imaged by a microscope objective (Newport, M-60x), relayed through a series of lenses, and focused on a CCD sensor (Point Grey, Chameleon). A liquid lens (Varioptic, Caspian) was used to manipulate the working distance of the system such that vessels at varying depths could be optically accessed without introducing motion artifact. A green-wavelength LED (λ = 532 nm) was used to locate vessels and a red-wavelength LED (λ = 655 nm) was used to detect Hz circulating within the superficial vessels. A block diagram of the MvM and representative images collected using a non-infected mouse are shown in Fig. 2. Blood samples were acquired immediately following each in vivo imaging session for thin film preparation. The presence of only iRBCs or pWBCs was confirmed by traditional blood smear microscopy. Videos collected from non-infected mice were used as negative controls.

**Fig. 3:**
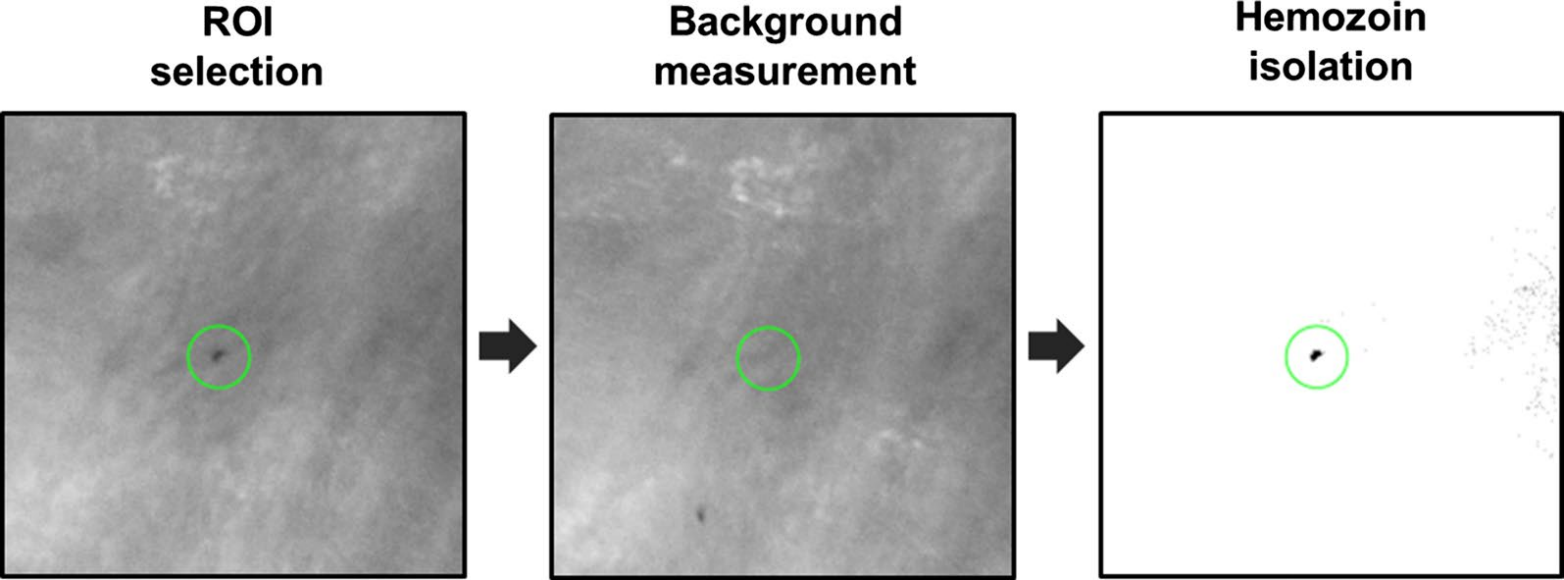
Measurements of haemozoin detected by in vivo microscopy. A region of interest (ROI) is centered on the haemozoin particle in a single frame from video collected in vivo (left). Time averaged background intensities were measured from the same ROI for frames before and after the selected frame to be analysed; an example background measurement is shown (middle). Averaged background measurements were used to generate a binary mask to isolate haemozoin signal from the selected frame to be analysed (right). The raw images that appear in this figure were contrast enhanced to allow for easier visualization of subcellular haemozoin particles

Videos collected under red light illumination were analysed in ImageJ. A 3 × 3 neighbourhood median filter was applied to each video to reduce noise. Hz particles were identified visually as dark structures against the light background within the vessel boundary. Only Hz particles that were easily discerned in a single frame without the aid of video playback and observed in at least five consecutive frames were analysed. A 15 μm diameter region of interest (ROI) was centered on the Hz particle in a single frame. Each ROI represents a single cell. Background intensity was measured for two frames before and after the selected frame to be analysed. Similar to blood smear microscopy analysis, the intensity threshold was selected as the average minimum background pixel intensity of the ROI over time. This threshold was applied to the ROI to isolate the Hz signal, as shown in the flow chart in Fig. 3. Hz area and pixel intensity were measured for each ROI to calculate Hz d_eff_ and RID respectively. Additionally, the particle velocity was measured over 5 frames. Measured Hz d_eff_ and RID were plotted for all ROIs by infection status—negative, acute (iRBCs), or post (pWBCs) infection—as determined by blood smear analysis. Box plots characterizing the first quartile, median, and third quartile with whiskers extending to ± 1.58 (IQR/(n^1/2^)) were generated for these data. Mann–Whitney rank sum tests were performed to measure whether differences in the median values of these parameters were statistically significant. ROC curves were generated using Hz d_eff_ and RID to discriminate between iRBCs and pWBCs and classify acute infection versus post infection. The area under the ROC curves was measured for comparison with classification performance in vitro of iRBCs and pWBCs.

## Results

### Feature identification using blood smear microscopy

Conventional microscopy of thin prep *P. yoelii*-infected blood smears was used to identify Hz-based features to differentiate between iRBCs and pWBCs. This method allowed for the investigation of Hz signal without confounding attributes intrinsic to in vivo imaging, such as flowing blood and tissue optical scattering. DAPI fluorescence nuclear staining was chosen to confirm cell type through morphology rather than traditional Giemsa vital dye staining. The DAPI molecule is excited under ultraviolet illumination and, therefore, does not interfere with the cell’s absorbance in the visible range. Cells were selected from colocalized images collected under narrow-band brightfield illumination (λ = 655 nm ± 20 nm) and a DAPI fluorescence filter set.

**Fig. 4:**
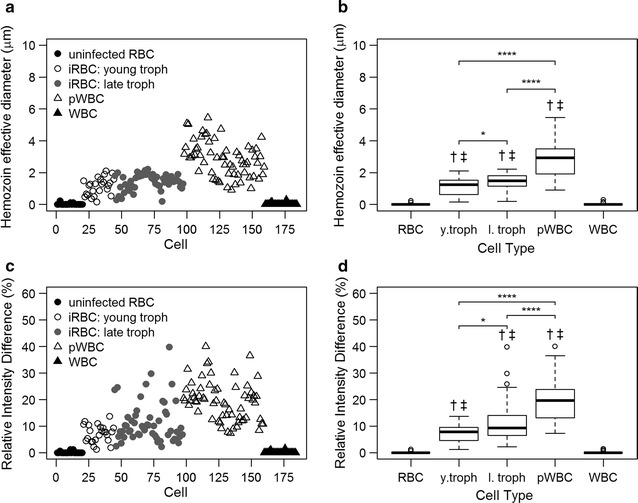
Haemozoin feature analysis of *Plasmodium yoelii* blood smears. Scatterplots of the measured Hz d_eff_ (**a**) and RID (**c**) values are shown. Box plots characterizing the first quartile, median, and third quartile of the Hz d_eff_ (**b**) and RID (**d**) data are plotted for each cell type: uninfected RBC, young trophozoite iRBC, late trophozoite iRBC, pWBC, and WBC. Median values of iRBCs and pWBCs were significantly different (****p ≤ 0.0001), independent of parasite maturity. Additionally, statistically significant differences between median values of young and late trophozoite iRBCs (*p ≤ 0.05) were observed. Significant differences (p ≤ 0.0001) of median values compared to negative control cell populations, uninfected RBCs (†) and WBCs (‡), were observed for all haemozoin-containing cell types

Hz d_eff_ and RID were measured for each cell and correlated with the morphologically determined cell type from the corresponding DAPI image. Scatter plots of Hz d_eff_ and RID grouped by cell type are shown in the left column of Fig. 4. The right column of Fig. 4 shows box and whisker plots of these parameters for each cell type. Significant differences between median values of cell types are indicated on these plots. Median (IQR) values of Hz deff were 1.24 (0.64–1.51) for young trophozoite iRBCs, 1.48 (1.14–1.80) for late trophozoite iRBCs, and 2.94 (1.94–3.49) for pWBCs. RID median values were 7.84 (4.67–9.43) for young trophozoites, 9.27 (6.56–14.10) for mature trophozoites, and 19.62 (13.18–23.70) for pWBCs. The median RID and Hz d_eff_ values of iRBCs were significantly different (p < 0.0001) from pWBCs. The median values of negative control cells, uninfected RBCs and pigment-free WBCs, were significantly different (p < 0.0001) from the Hz-containing cells, iRBCs and pWBCs, for both parameters. Additionally, the RID and Hz d_eff_ median values were significantly greater for late trophozoites than young trophozoites (p < 0.05), suggesting the potential to estimate parasite maturity using these features.

**Fig. 5:**
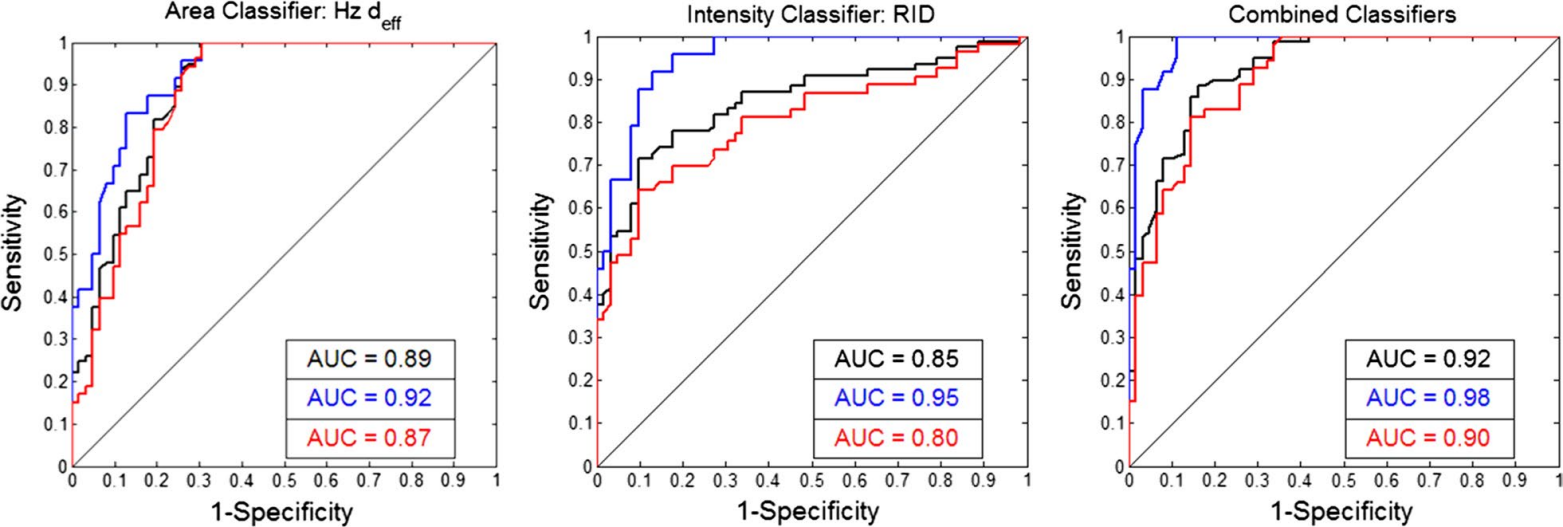
ROC curves for the classification of iRBCs and pWBCs in *P. yoelii* blood smears. Linear discriminant analysis was used to develop an algorithm to classify iRBCs and pWBCs based on the measured haemozoin feature values; corresponding ROC curves (black line) are shown for algorithms based on Hz d_eff_ (left), RID (middle), and the combined feature (right). The area under the curve (AUC) increased when late trophozoites were removed from the analysis (blue line). The AUC decreased when young trophozoites were removed from the data set and only late trophozoites were included in the analysis (red line)

Figure 5 shows the ROC curves for an algorithm to discriminate between iRBCs and pWBCs using Hz d_eff_ and RID, alone and in combination. The area under the curve (AUC) for Hz d_eff_ and RID were 0.89 and 0.85, respectively. The AUC for the combined parameters was 0.92. It is important to note that all levels of parasite maturity are readily observed during *P. yoelii* infection, however in *P. falciparum* infection sequestration of mature parasites decreases the number of mature iRBCs found in peripheral blood circulation. If late trophozoites are removed from the data set, the AUC increases to 0.92, 0.95, and 0.98, for each parameter respectively. Conversely, if young trophozoites are removed from the data set and only late trophozoites are compared to pWBC, the AUC decreases to 0.87, 0.80, and 0.90 for Hz d_eff_, RID, and the combination of the two respectively.

Because of the wide variation in reported pWBC concentration, it is important to consider the relative ratio of iRBC to pWBC when assessing the ability to discriminate by cell type. For example, in all the cases reported in Additional file 1: Table S1, parasitaemia is at least eight times greater than pWBC concentration. The AUC was measured for ten randomly selected data sets with a relative ratio of 10:1 iRBC to pWBC in each set, yielding AUC values ranging from 0.73 to 1.00 for Hz d_eff_, 0.73 to 0.92 for RID, and 0.75 to 1.00 for the combined metrics. Similarly, the AUC was measured for ten randomly selected data sets with a relative ratio of 1:10 iRBC to pWBC in each set, achieving AUC values ranging from 0.81 to 0.95, 0.64 to 0.97, and 0.80 to 0.99 for Hz d_eff_, RID, and the combined classifiers, respectively.

### In vivo discrimination of Hz signal

**Fig. 6:**
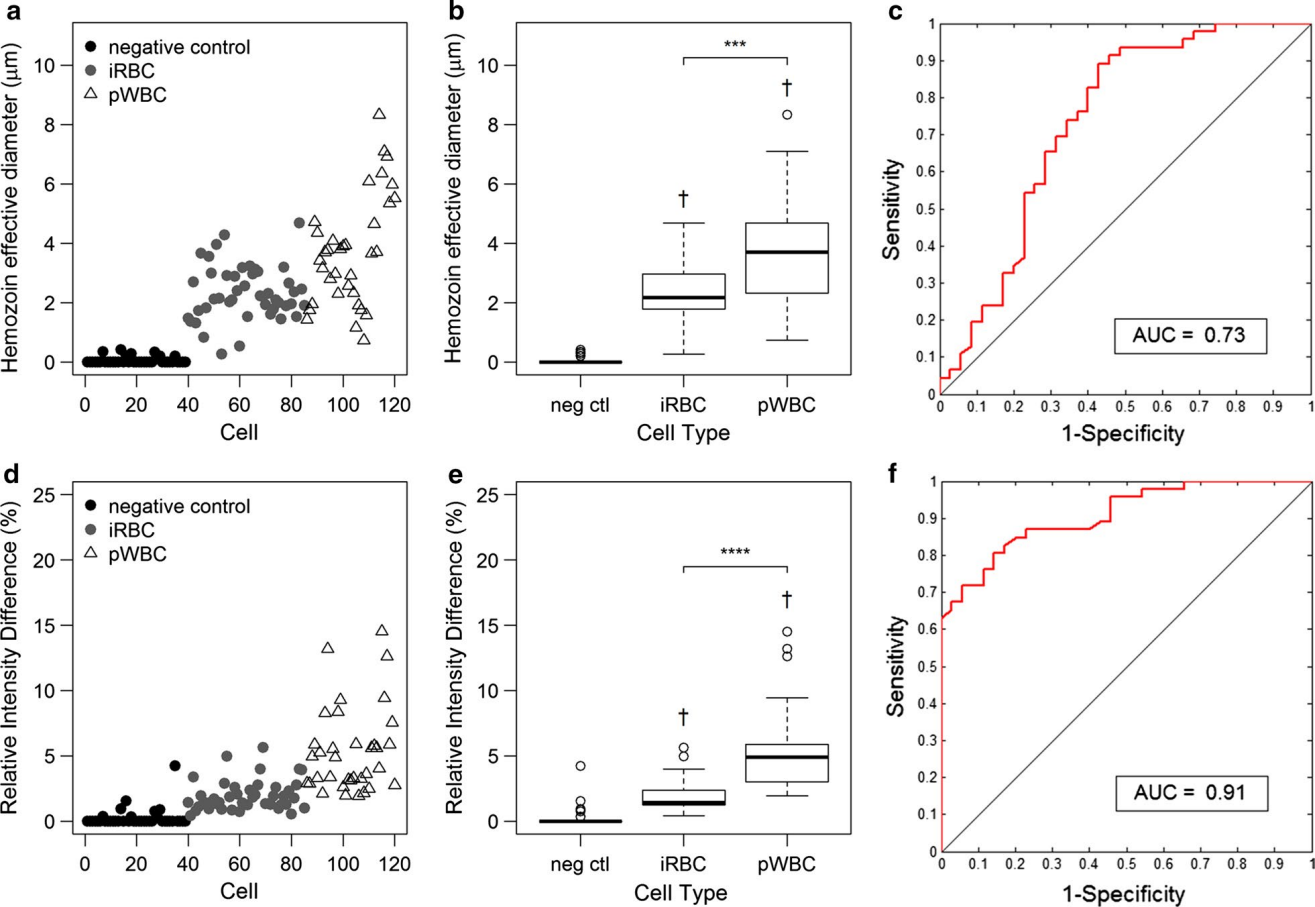
Haemozoin absorbance signal features from *P. yoelii* infection measured in vivo. Scatterplots are shown for the size-based feature Hz d_eff_ (**a**) and the intensity-based feature RID (**d**). The box plots of the median, first, and third quartile data are shown for each feature (**b**, **e**). Significant difference between the median iRBC and pWBC values are indicated by *** (p ≤ 0.001) and **** (p ≤ 0.0001). Significant difference (p ≤ 0.0001) between the median values of the experimental cohort and negative control is indicated by †. The classification performance of each feature is shown using ROC curves (**c**, **f**)

The Hz signal features identified in blood smear microscopy were evaluated in a mouse model to classify cell type based on analysis of images of circulating cells acquired in vivo. Prior to imaging, subjects were categorized as current acute infection having iRBCs only or as post infection with pWBCs only, confirmed by blood smear microscopy. Circulating Hz was visually observed under red light illumination as small dark particles flowing through the blood vessel. The intensity and area of each particle was measured and used to calculate the Hz d_eff_ and RID. Randomly selected ROIs from uninfected subjects were used as negative controls. Hz d_eff_ and RID values measured in vivo are plotted in the left column of Fig. 6. These data show a similar trend to the blood smear data, with larger Hz d_eff_ and RID values for pWBCs in post infected animals as compared to iRBCs in animals with acute infections. Box and whisker plots of these parameters are shown in the middle column of Fig. 6. Median (IQR) values of Hz d_eff_ measurements were 2.17 (1.79–2.94) for iRBCs and 3.70 (2.31–4.68) for pWBCs. RID median values were 1.44 (1.25–2.36) for iRBCs and 4.90 (3.02–5.89) for pWBCs. Significant difference between median values of iRBCs and pWBCs were observed for both Hz d_eff_ and RID, p < 0.001 and p < 0.0001, respectively. The right side of Fig. 6 shows the ROC curve for an algorithm to differentiate between iRBCs and pWBCs based on these parameters. In vivo, RID performed better than Hz d_eff_ as a classifying feature, with an AUC of 0.91 and 0.73, respectively. Combination of the features resulted in an AUC of 0.83. As seen in the analysis of the *P. yoelii*-infected blood smear data, the relative ratio of iRBCs to pWBCs will affect the AUC. For a relative ratio of 1:10 iRBC to pWBC, the AUC varies from 0.61 to 0.85 for Hz d_eff_, 0.79 to 1.00 for RID, and 0.84 to 0.90 for the combined classifiers in vivo. For a relative ratio of 10:1 iRBC to pWBC, the AUC varies from 0.27 to 0.99 for Hz d_eff_, 0.78 to 0.99 for RID, and 0.54 to 0.91 for the combined classifiers. Variability in the Hz d_eff_ measurements may arise from cells coming in and out of the focal plane as they translate through the vessel. Additionally, only pWBCs occurring post-infection were investigated in vivo; the Hz d_eff_ may be larger in pWBCs present during an active infection when more Hz is available to be phagocytized.

**Fig. 7:**
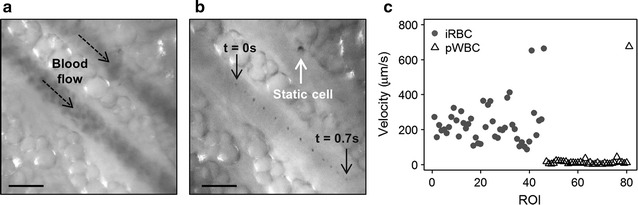
Cell velocity measured in vivo. **a** Blood flow detected under green light illumination (λ = 532 nm). **b** Successive frames collected under red light illumination (λ = 655 nm) are overlaid to show a single haemozoin particle tracked through the vessel over time. **c** Average velocities of iRBCs detected during acute infection are higher than velocities of pWBCs measured post infection. Scale bars = 20 μm

The particle velocity is another feature of the Hz signal which is readily measured in vivo. This is illustrated in an overlay of successive frames from a video captured under red-light illumination in Fig. 7b. Here, a single Hz particle is tracked as it travels through the bottom vessel. In the vessel at the top of the frame, a static cell does not move over the period of 0.7 s, suggesting cytoadhesion to the endothelial wall. The same FOV imaged under green-light illumination is shown to clarify vessel location in the red illumination overlay (Fig. 7a). Average cell velocities were measured over five consecutive frames for each ROI; results are shown in the scatterplot in Fig. 7c. There is a distinct separation between the slow moving pWBCs and faster circulating iRBCs. This information could be helpful to indicate cases of cytoadhesion and sequestration in vivo and shows promise as a potential classification feature to be validated in human subjects.

### Evaluation of *Plasmodium falciparum* Hz signal

*Plasmodium falciparum*-infected blood smears were used to evaluate the Hz signal discriminating features in a human infecting species of malaria parasites. Although these samples provided iRBCs for analysis, pWBCs were not readily detected in the smears. The lack of pWBC observation may be due to the timing of infection or very low concentrations of pWBCs. Hz is only phagocytized by neutrophils and monocytes, which account for a fraction of the total number of blood cells in a microlitre of blood; for comparison, typical concentrations of neutrophils range from 1800/µl to 7000/µl and monocytes range from 100/µl to 800/µl, whereas the concentrations of RBCs range from 4.0 to 5.5 million/µl [18], Also, the fraction of pWBCs observed in malaria infected individuals varies widely [19]. To investigate the Hz signal in pWBCs, phagocytosis of Hz isolated from *P. falciparum* cultured samples was induced in whole blood collected from healthy human donors. After exposure to Hz, thin smears were prepared from the blood samples and examined by conventional microscopy. DAPI signal was again used to differentiate cell type by nuclear morphology.

**Fig. 8:**
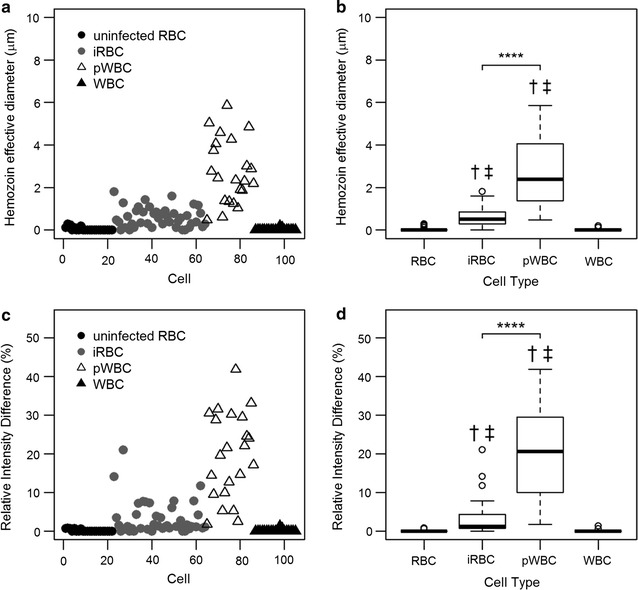
Haemozoin feature analysis of *P. falciparum* samples. Scatterplots for the Hz d_eff_ feature (**a**) and RID (**c**) are shown. Corresponding box plots are shown for each feature (**b**, **d**). Significant separation was observed between median values of iRBCs and pWBCs as indicated by **** (p ≤ 0.0001). Significant difference (p ≤ 0.0001) compared to median values of uninfected RBCs and WBCs are indicated by † and ‡, respectively

The Hz d_eff_ and RID measured in *P. falciparum* blood smears are plotted by cell type in the left column of Fig. 8. Only immature iRBCs were detected in the patient-derived smears, consistent with sequestration of mature iRBCs in *P. falciparum* pathology. Box and whisker plots of these parameters are shown in the right column of Fig. 8. The median (IQR) values of Hz d_eff_ were 0.52 (0.28–0.86) for iRBCs and 2.40 (1.50–3.98) for pWBCs. RID median values were 1.27 (0.72–4.30) for iRBCs and 20.60 (10.63–29.33) for pWBCs. The differences between the median values of iRBCs and pWBCs were statistically significant (p < 0.0001) for both RID and Hz d_eff_. The median values of the negative control cells without Hz (uninfected RBCs and WBCs) were also significantly different from all other groups for both parameters.

**Fig. 9:**
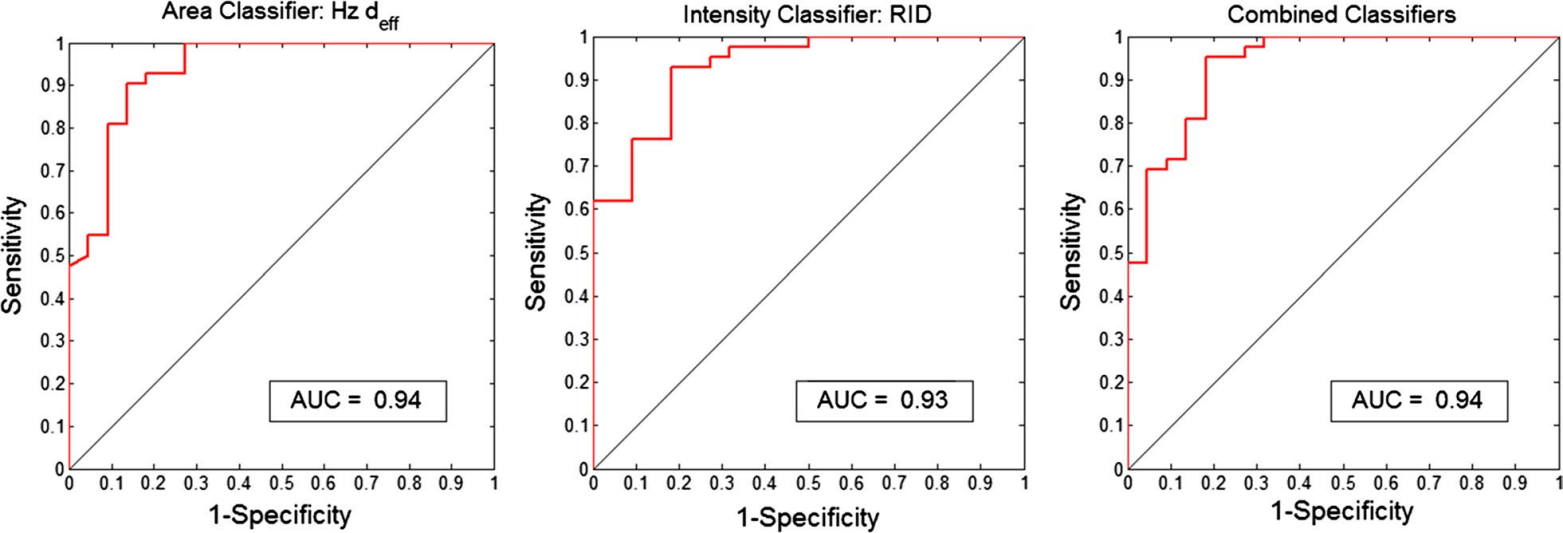
ROC curves for the classification of iRBCs and pWBCs in *P. falciparum* blood smears. ROC curves and the AUC are shown for Hz d_eff_ (left), RID (middle), and the combination of the two features (right)

These features were used to discriminate between iRBCs and pWBCs; resulting ROC curves are shown in Fig. 9. The AUC for Hz d_eff_ and RID were 0.94 and 0.93 respectively. When these features are combined the AUC is 0.94. These values are similar to those obtained using these features to discriminate between young trophozoite iRBCs and pWBCs from the *P. yoelii* blood smear data. AUCs were measured for ten randomly selected data sets with a relative ratio of 1:10 iRBCs to pWBC, yielding AUCs ranging from 0.89 to 0.99 for Hz d_eff_, 0.86 to 1.00 for RID, and 0.88 to 0.99 for the combined classifiers. When the relative ratio was set to 10:1 iRBC to pWBC the AUC ranged from 0.74 to 1.00, 0.80 to 1.00, and 0.74 to 1.00 for Hz d_eff_, RID, and the combined classifiers, respectively.

## Discussion

Diagnosis of malaria based on symptoms alone suffers from poor specificity, leading to inadequate treatment of patients, over-use of anti-malarial drugs and ultimately drug resistance. The advent of rapid diagnostic tests has assisted clinicians in making accurate diagnoses, but for these tests to be impactful, it is critical that they be cost effective, easy to use, and ultimately improve the specificity of clinical diagnosis [20]. The use of in vivo microscopy to rapidly detect malaria in vivo without the need for a blood sample or associated consumables was recently reported [1]. This method relies on the endogenous malaria biomarker haemozoin (Hz) for detection of malaria parasites, making the test simple but presenting the potential for false positives with the presence of phagocytized Hz in white blood cells that persist after an infection has cleared. Differentiating iRBCs and pWBCs is especially important in endemic populations that are reported to have observed baseline pWBC presence in the blood of healthy subjects [5, 8].

In this study, the clinical utility of in vivo Hz detection is furthered by identifying a simple metric to discriminate iRBCs from pWBCs. *Plasmodium yoelii*-infected mouse blood smears were analysed to assess Hz size and intensity-based features, Hz d_eff_ and RID, respectively, for the potential to classify iRBCs from pWBCs. When combined, these features yielded an algorithm with an AUC of 0.92. These features were then assessed in vivo in a mouse model of malaria. Investigations in vivo add complexity to the measurement of Hz signal features. Hz signal is detected within moving cells found in vessels at varying depths below the tissue surface. The potential for image distortion is increased with the addition of motion artifacts and tissue scattering. Despite the added complexity of in vivo detection, RID demonstrated similar classification potential as seen in the blood smear analysis, suggesting that the features identified using blood smear microscopy can be implemented to classify cell type by Hz signal detected in vivo.

*Plasmodium falciparum* blood samples were used to evaluate the Hz d_eff_ and RID classification features in Hz generated by human infecting strains of *Plasmodium*. Multinucleated parasites were not observed in these samples, likely due to sequestration of mature iRBCs. Physical attributes, such as size and optical absorbance, are expected to be smaller for Hz in immature iRBCs as the parasite has had less time to generate Hz. The absence of mature iRBCs and therefore larger Hz structures allows for even greater differences between the measured Hz features of iRBCs and pWBCs. This effect may be seen in the classification performance of the *P. falciparum* samples, achieving an AUC of 0.94 and 0.93 for Hz d_eff_ and RID, respectively. These results confirm the potential to discriminate by cell type in human infecting species using Hz absorbance signal.

The ability to identify cell type reduces the potential for false positive Hz-based malaria diagnosis. Other techniques that use Hz as a biomarker are limited by the lack of a method to discriminate iRBCs from pWBCs. Here, it is demonstrated for the first time that simple features of the Hz absorbance signal, such as size and intensity, may be used to discriminate between iRBCs and pWBCs. This classification approach could be extended to other Hz detection methods that measure similar physical parameters, such as photoacoustic or polarization based approaches. For example, one study suggests that there is a positive correlation between the depolarization signal intensity and parasite maturity [17]. Another method using photo-induced acoustic signals to detect Hz reported an increased acoustic trace amplitude of schizonts compared to ring form parasites [3].

In this study, the approach of in vivo Hz detection was assessed using a very simple and low cost microscope platform. One potential obstacle to the implementation of an image analysis-based classification algorithm is the need for an electronic computational platform such as computer or cell phone to measure the features. Another consideration for future implementation of Hz based cell discrimination is the addition of other metrics that are discernable in vivo, such as cytoadhesion and velocity. These measurable and readily observable features could give valuable information about disease severity and host immune response [21].

Both neutrophils and monocytes of the host’s white blood cell population phagocytize Hz. There is differing clinical significance and meaning associated with the presence of each of these pWBCs. For example, the presence of pigment-containing neutrophils could indicate a severe infection whereas pigment-containing monocytes could signal severe anaemia [5, 6]. Additionally, the lifetimes of these two cell types differ greatly, on the order of days for neutrophils compared to over a week for monocytes. Due to their long lifespans, monocytes persist in the bloodstream longer and pose a greater risk for generating a false positive in a Hz based test. Therefore, future investigations into further classifying cell type to distinguish between granulocytes and agranulocytes should be considered.

## Conclusion

This work describes a method to discriminate between Hz originating from iRBCs or pWBCs in a needle-free malaria diagnostic test. This approach addresses one of the key potential shortcomings of a Hz-based diagnostic by reducing the potential for false positives due to the persistence of Hz found in pWBCs. Two intrinsic Hz signal properties, pixel intensity and size, were evaluated by blood smear microscopy as classification features for cell type differentiation. Using blood smear microscopy of two species of *Plasmodium*, the Hz RID and d_eff_ were identified as measurable features to distinguish iRBC from pWBC. Further analysis showed that Hz RID specifically showed greatest potential for the differentiation of iRBC and pWBC in vivo. Ultimately, the addition of cell-type discrimination makes this Hz-based malaria diagnostic technique more robust, increasing its clinical utility.

## Additional file

**Additional file 1: Table S1.** Summary of reported pWBC concentrations in *Plasmodium falciparum* infection. Median and range values of pigment-containing neutrophils and pigment-containing monocytes reported in the literature are listed. Interquartile range values are indicated (a); all other range values represent the minimum and maximum reported values. Italicized font indicates values were converted from reported percentage to #/µl assuming 5500 neutrophils/µl and 500 monocytes/µl. Disease severity, study population age group and location are given for each source. Mean (b) or median (c) reported parasitaemia values are also listed.

## Abbreviations

Hz: haemozoin
ROI: region of interest
RBC: red blood cell
iRBC: infected red blood cell
WBC: white blood cell
pWBC: pigment-containing white blood cell
RID: relative intensity difference
PBS: polarized beam splitter
LP: linear polarizer
CL: collimating lens
ROC: receiver operating characteristic
AUC: area under the curve
d_eff_: effective haemozoin diameter
MvM: microvascular microscope
IQR: interquartile ratio

## References

[1] Burnett JL, Carns JL, Richards-Kortum R (2015). In vivo microscopy of hemozoin: towards a needle free diagnostic for malaria. Biomed Opt Express.

[2] Newman DM, Heptinstall J, Matelon RJ, Savage L, Wears ML, Beddow J (2008). A magneto-optic route toward the in vivo diagnosis of malaria: preliminary results and preclinical trial data. Biophys J.

[3] Lukianova-Hleb EY, Campbell KM, Constantinou PE, Braam J, Olson JS, Ware RE (2014). Hemozoin-generated vapor nanobubbles for transdermal reagent- and needle-free detection of malaria. Proc Natl Acad Sci USA.

[4] Mujuzi G, Magambo B, Okech B, Egwang TG (2006). Pigmented monocytes are negative correlates of protection against severe and complicated malaria in Ugandan children. Am J Trop Med Hyg.

[5] Lyke KE, Diallo DA, Dicko A, Kone A, Coulibaly D, Guindo A (2003). Association of intraleukocytic *Plasmodium falciparum* malaria pigment with disease severity, clinical manifestations, and prognosis in severe malaria. Am J Trop Med Hyg.

[6] Day NP, Pham TD, Phan TL, Dinh XS, Pham PL, Ly VC (1996). Clearance kinetics of parasites and pigment-containing leukocytes in severe malaria. Blood.

[7] Metzger WG, Mordmüller BG, Kremsner PG (1995). Malaria pigment in leukocytes. Trans R Soc Trop Med Hyg.

[8] Amodu OK, Adeyemo AA, Olumese PE, Gbadegesin RA (1998). lntraleucocytic malaria pigment and clinical severity in children. Trans R Soc Trop Med Hyg.

[9] Hänscheid T, Längin M, Lell B, Pötschke M, Oyakhirome S, Kremsner PG (2008). Full blood count and haemozoin-containing leukocytes in children with malaria: diagnostic value and association with disease severity. Malar J.

[10] Lell B, Missinou MA, Issifou S, Matsiegui P-B, Olola CHO, Taylor TE (2005). Assessment of a simplified method for counting leukocytic malaria pigment. Am J Trop Med Hyg.

[11] Phu NH, Day N, Diep PT, Ferguson DJP, White NJ (1995). Intraleucocytic malaria pigment and prognosis in severe malaria. Trans R Soc Trop Med Hyg.

[12] Grande R, Boschetti C (2011). Intracytoplasmic hemozoin (malarial pigment) in a case of severe, aparasitemic *Plasmodium falciparum* malaria. Am J Trop Med Hyg.

[13] Menendez C, Ordi J, Ismail MR, Ventura PJ, Aponte JJ, Kahigwa E (2000). The impact of placental malaria on gestational age and birth weight. J Infect Dis.

[14] Chua CLL, Robinson LJ, Baiwog F, Stanisic DI, Hamilton JA, Brown GV (2015). High numbers of circulating pigmented polymorphonuclear neutrophils as a prognostic marker for decreased birth weight during malaria in pregnancy. Int J Parasitol.

[15] Thomson-Luque R, Scopel KKG (2015). Immature reticulocytes as preferential host cells and the challenges for in vitro culture of *Plasmodium vivax*. Pathog Glob Health.

[16] Hyman BC, Macinnis AJ (1979). Rapid detection of malaria and other bloodstream parasites by fluorescence microscopy with 4′6 diamidino-2-phenylindole (DAPI). J Parasitol.

[17] Frita R, Rebelo M, Pamplona A, Vigario AM, Mota MM, Grobusch MP (2011). Simple flow cytometric detection of haemozoin containing leukocytes and erythrocytes for research on diagnosis, immunology and drug sensitivity testing. Malar J.

[18] McKenzie SB, Williams JL. Clinical Laboratory Hematology. Second. Zeiberg E, editor. Upper Saddle River, New Jersey: Pearson; 2010.

[19] Hänscheid T, Egan TJ, Grobusch MP (2007). Haemozoin: from melatonin pigment to drug target, diagnostic tool, and immune modulator. Lancet Infect Dis.

[20] Rafael MEM, Taylor T, Magill A, Lim YY-W, Girosi F, Allan R (2006). Reducing the burden of childhood malaria in Africa: the role of improved. Nature.

[21] Dondorp AM, Ince C, Charunwatthana P, Hanson J, van Kuijen A, Faiz MA (2008). Direct in vivo assessment of microcirculatory dysfunction in severe falciparum malaria. J Infect Dis.

