# Supplementary material for: Towards a needle-free diagnosis of malaria: in vivo identification and classification of red and white blood cells containing haemozoin
Source: Malar J. 2017 Nov 7;16:447. doi: 10.1186/s12936-017-2096-1 (PMC5678583; doi:10.1186/s12936-017-2096-1)
Supplement: Supplementary file 1 — Additional file 1: Table S1. Summary of reported pWBC concentrations in Plasmodium falciparum infection. Median and range values of pigment-containing neutrophils and pigment-containing monocytes reported in the literature are listed. Interquartile range values are indicated (a); all other range values represent the minimum and maximum reported values. Italicized font indicates values were converted from reported percentage to #/µl assuming 5500 neutrophils/µl and 500 monocytes/µl. Disease severity, study population age group and location are given for each source. Mean (b) or median (c) reported parasitaemia values are also listed. [file 12936_2017_2096_MOESM1_ESM.docx]

| Pigmented Neutrophils (μl^-1^) | Pigmented Monocytes (μl^-1^) | Disease Severity | Study Population | Location | Parasitemia (μl^-1^) | Reference |
| --- | --- | --- | --- | --- | --- | --- |
| 0 (0 – 80) | 32 (0 – 640) | severe | children | Uganda | 33,980^c^ | Mujuzi^4^ |
| 0 (0 – 16) | 0 (0 – 272) | uncomplicated | children | Uganda | 1,560^c^ | Mujuzi^4^ |
| 349 (0 – 3,721) | 216 (0 – 3,420) | severe | children | Mali | 174,428^c^ | Lyke^5^ |
| 64 (0 – 534) | 94 (0 – 1,698) | uncomplicated | children | Mali | 8,200^c^ | Lyke^5^ |
| 165(0 – 1,540) | 100 (0 – 316) | severe | adult | Vietnam | 90,500^c^ | Day^6^ |
| *110 (0 – 825)* | *120 (10 – 285)* | severe | children | Gabon | 91,300^c^ | Metzger^7^ |
| *0 (0 – 385)* | *35 (0 – 225)* | uncomplicated | children | Gabon | 20,600^c^ | Metzger^7^ |
| *0 (0 – 55)* | *5 (0 – 105)* | uncomplicated | adult | Gabon | 8,600^c^ | Metzger^7^ |
| *1485 (825 – 2,090)^a^* | *265 (185 – 350) ^a^* | severe | children | Nigeria | 14,454^b^ | Amodu^8^ |
| *495 (220 – 935) ^a^* | *85 (65 – 150) ^a^* | uncomplicated | children | Nigeria | 6,561^b^ | Amodu^8^ |

**Additional Table S1. Summary of reported pWBC concentrations in *P. falciparum* infection.** Median and range values of pigment-containing neutrophils and pigment-containing monocytes reported in the literature are listed. Interquartile range values are indicated (a); all other range values represent the minimum and maximum reported values. Italicized font indicates values were converted from reported percentage to #/µl assuming 5500 Neutrophils/µl and 500 monocytes/µl. Disease severity, study population age group and location are given for each source. Mean(b) or median(c) reported parasitemia values are also listed.
